# Micro-phenomenology of immersion and perceived presences under DMT

**DOI:** 10.1093/nc/niag015

**Published:** 2026-07-14

**Authors:** James W Sanders, Raphaël Millière, Ema Demšar, Zachary G Daily, David Erritzoe, Robin Carhart-Harris, Christopher Timmermann

**Affiliations:** UCL Centre for Consciousness Research, Department of Experimental Psychology, University College London, 26 Bedford Way, London WC1H 0AP, United Kingdom; DMT Research Group, Centre for Psychedelic Research, Department of Brain Science, Imperial College London, Burlington Danes, The Hammersmith Hospital, Du Cane Road, London W12 0NN, United Kingdom; Faculty of Philosophy, University of Oxford, The Stephen A. Schwarzman Centre for the Humanities, Radcliffe Observatory Quarter, Woodstock Road, Oxford, OX2 6GG, United Kingdom; Monash Centre for Consciousness and Contemplative Studies, Department of Philosophy, Monash University, 29 Ancora Imparo Way, Monash University, Clayton Campus, Victoria 3168, Australia; UCL Centre for Consciousness Research, Department of Experimental Psychology, University College London, 26 Bedford Way, London WC1H 0AP, United Kingdom; Center for Psychedelic and Consciousness Research, Department of Psychiatry and Behavioral Sciences, Johns Hopkins School of Medicine, Joseph V. Brady Behavioral Biology Research Building, Johns Hopkins Bayview Medical Center, 5510 Nathan Shock Drive, Baltimore, MD 21224, United States; UCL Centre for Consciousness Research, Department of Experimental Psychology, University College London, 26 Bedford Way, London WC1H 0AP, United Kingdom; UCL Centre for Consciousness Research, Department of Experimental Psychology, University College London, 26 Bedford Way, London WC1H 0AP, United Kingdom; Department of Neurology, University of California San Francisco, 400 Parnassus Ave, 8th Floor, San Francisco, CA 94143, United States; UCL Centre for Consciousness Research, Department of Experimental Psychology, University College London, 26 Bedford Way, London WC1H 0AP, United Kingdom; DMT Research Group, Centre for Psychedelic Research, Department of Brain Science, Imperial College London, Burlington Danes, The Hammersmith Hospital, Du Cane Road, London W12 0NN, United Kingdom

**Keywords:** DMT, micro-phenomenology, neurophenomenology, immersion, perceived presences, altered states of consciousness, multisensory integration, psychedelics

## Abstract

Psychedelic compound N,N-Dimethyltryptamine (DMT) is renowned for inducing highly immersive experiences, often including encounters with seemingly sentient presences. While such phenomena are well documented, immersion under psychedelics remains conceptually underdefined and phenomenologically underexplored. Here we apply micro-phenomenology to characterize immersion under DMT as a structured continuum from subtle to gross forms of immersion, and to examine how perceived presences arise within this continuum. Twenty-three participants received 20 mg intravenous DMT during simultaneous fMRI–EEG acquisition, followed by detailed micro-phenomenological interviews focused on the temporal unfolding of experience. Micro-phenomenological analysis methods yielded 125 phenomenological categories describing structural dimensions, including sensory and amodal faculties, spatial organisation, self–world configuration, and modes of social relatedness. Dynamic analysis revealed reliable developmental patterns, with bodily effects typically preceding visual and auditory ones, and perceived presences emerging only after multisensory integration and 3D spatial characteristics had developed, illustrating a hierarchical relationship between subtle and gross forms of immersion. Perceived presences varied widely in sensory modality, semantic complexity, and relational mode. These findings show immersion under DMT as a dynamic, constructive process, providing precise structural features for future neurophenomenological modelling, and supporting the view that DMT research may inform our understanding of immersive experience across other aetiologies, including ordinary experience of the world.

Highlights

- Micro-phenomenology mapped immersive structure in the DMT experience
- Immersion unfolds along a structured continuum from subtle to gross forms
- Multisensory and spatial integration precede experiences of perceived presences
- Perceived presences emerge as high-order features of immersive experience

## Introduction

N,N-Dimethyltryptamine (DMT) is a serotonergic psychedelic, increasingly used as a perturbational tool for researching brain mechanisms of conscious experience (Timmermann, Roseman, et al. 2023, Timmermann et al. 2019) and for its potential benefits to mental health (Timmermann et al. 2024, Falchi-Carvalho et al. 2025). DMT is commonly found across plant and animal life, and is a key psychoactive component of Ayahuasca, a psychedelic plant admixture used by indigenous tribes of the Amazon rainforest for a variety of ritual purposes (Labate and MacRae 2016).

DMT is notable for the intensity and richness of the experiences it produces. Previous research shows that it induces striking experiences of complex, other worlds, sometimes seemingly inhabited by sentient beings (henceforth: perceived presences), as well as radical alterations to the experience of self (Strassman 1996, Michael et al. 2021, Michael et al. 2023). This profound shift in the content and structure of experience, often accompanied by a diminished awareness of the external environment, suggests that DMT may be a useful tool to investigate the deconstruction of ordinary experience and the reconstruction of an alternative experience, rich in sensory, emotional, and spatial contents (Lawrence et al. 2022, Timmermann, Roseman, et al. 2023).

The reconstructed experience under DMT has been characterized as immersive (Timmermann, Roseman, et al. 2023, Michael et al. 2021), however immersion under psychedelics has not yet been explicitly defined or explored. Previous questionnaire and interview studies have coarsely described diverse sensory, cognitive, affective, and metacognitive dimensions (Davis et al. 2020, Michael et al. 2021, 2023, Lawrence et al. 2022), and identified characteristic DMT experiences of sensory disconnection from the environment, and entering emergent ‘world-analogues’ and engaging with perceived presences (Shanon 2002, Strassman et al. 2008, Timmermann et al. 2019). Therefore, we took these phenomena of sensory disconnection, and interaction or involvement with a world-analogue, as a starting point in this phenomenological exploration of immersion under DMT. Additionally, perceived presences—which feature in a range of disordered and non-ordinary states, including psychosis, Parkinson’s, Charles Bonnet syndrome, spiritualism, sleep paralysis and endurance sports (Sacks 2013, Barnby et al. 2023)—are useful for the study of social cognition and its neurobiological mechanisms. However, a phenomenological investigation of immersion and how it relates to perceived presences in the DMT experience has not been conducted.

Previous phenomenological research into DMT has prioritized the *content* of the experience (Davis et al. 2020, Michael et al. 2021, 2023, Lawrence et al. 2022), however, it has been argued that prioritising the *structure* of experience (including its *dynamic processes*) in research can better contribute to the study of consciousness (Timmermann, Bauer, et al. 2023, Varela 1996). There are grey areas between what can be considered content *versus* structure, however we can generally distinguish content as the variable, identified elements or objects that a DMT experience is *about* (e.g. a jester-like entity in a jewel encrusted arena), whereas structure refers to the relatively invariant aspects of experience that structure *how contents are experienced.* These include the sensory modalities, attentional modes, features of the sense of self, time and space, as well as the interrelations between these features (e.g. was the jester and its environment represented visually or otherwise? Did it have a particular spatial configuration? From which perspective was it experienced?). Preliminary phenomenological observations showing the potential of a structural focus in DMT research have yielded novel hypotheses pertaining to specific spatiotemporal forms and constants in the visual field under DMT (Gómez-Emilsson 2016), however their generalisability has not been tested, and many other structures remain unstudied. We regard immersion to be a structural quality that describes *how* the subject is relating to experience and its contents, regardless of *what* specific content is experienced, and so a structural research approach is especially relevant.

Furthermore, studies of psychedelic experience have traditionally been limited. Popular methods often lack temporal and phenomenological precision. Questionnaires obscure dynamic processes by averaging across entire experiences, and rely on broad constructs like ‘mystical experience’ or ‘ego dissolution,’ which may reflect theoretical bias rather than lived experience (Timmermann, Bauer, et al. 2023, Taves 2020, Millière et al. 2018, Sanders and Zijlmans 2021). While qualitative methods offer openness to participants’ own words, they also present challenges, such as recall errors, post-hoc rationalisation, and demand characteristics (Nisbett and Wilson 1977, Lush et al. 2020). Therefore, biases, confabulation, and other common threats to reliability and validity of descriptions of experience should be methodologically accounted for (Depraz et al. 2003), but for most qualitative methods this is arguably not the case.

Preliminary work shows that issues of phenomenological and temporal precision may be addressed using micro-phenomenology (Petitmengin et al. 2013), whereby research participants are guided by a trained interviewer to immerse themselves in the memory of an experience and describe it in a relatively refined and comprehensive way (Petitmengin 2006). The micro-phenomenological interview and analysis method are tailored for identifying coherent timelines of experience, and discouraging and excluding generalized statements that do not concern a specific moment of experience (Petitmengin et al. 2019, Valenzuela-Moguillansky and Vásquez-Rosati 2019). An early application of micro-phenomenology to psychedelic research studied visual, bodily, emotional, and metacognitive dimensions of DMT experience, comparing phenomenological changes over time to acute state brain activity (Timmermann et al. 2019). This research validated the use of micro-phenomenology in temporally sensitive, neurophenomenological, psychedelic research (neurophenomenology being a paradigm in cognitive neuroscience under which first- and third- person approaches to study the mind and brain are treated with equal importance and rigor, under the presumption that they mutually constrain so that each informs the other; Varela 1996).

Expanding on previous research, the present study employs the micro-phenomenological method to study immersion and perceived presences under DMT in a lab setting. Our study was guided by the following exploratory research questions: what are the structural dimensions on which the phenomenology of immersion and perceived presences under DMT unfolds? How do these dimensions relate to each other dynamically as this unfolding occurs? Our aim was to identify and characterize these structures to inform future comparative and neurophenomenological research on DMT and other altered states of consciousness.

## Methods

### Study design

This study is part of a broader neurophenomenological investigation utilising a single blind, between-subjects, placebo-controlled design. Participation consisted of two separate testing days, a minimum of two weeks apart. On each testing day, participants were screened for alcohol and other drugs of abuse before undergoing two testing sessions: in one session, they received IV DMT (20 mg DMT fumarate dissolved in 10 ml sterile saline), in the other session, they received IV placebo (10 ml sterile saline), in counterbalanced order. During testing sessions, participants lay in the scanner bed, blindfolded, with eyes closed. A combination of functional magnetic resonance imaging (fMRI) and electroencephalography (EEG) was recorded for 8 minutes prior, and 20 minutes subsequent, to placebo/DMT administration (more details of the neuroimaging component can be found in Timmermann, Roseman, et al. 2023). The first session of each scanning day was ‘task free,’ and, for the second session of the day, subjective ratings of drug intensity were given verbally by participants in response to audio cues every minute during scanning. Following the second session, a micro-phenomenological interview was performed, covering the experience of that session only. Thus, for the purposes of this study, the interview and experience sampling data from the second scanning session (where DMT was given) was used.

### Participants

25 healthy participants were recruited by word of mouth (10 female, mean age = 33.4 y, SD = 7.9). Two participants dropped out prior to the session involving the micro-phenomenological interview (final sample: 23 participants, eight female, mean age = 33.4 y, SD = 8.3). Interview data for two participants was incomplete due to technical issues. Participants were medically screened for conditions including heart disease, epilepsy, or a family history of psychotic disorder. Other exclusion criteria included: < 18 years old, psychedelic naivete, MRI contraindications, and excessive use of alcohol and/or other drugs of abuse. Participants gave written, informed consent before commencing participation. The study was conducted under the guidelines of the revised Declaration of Helsinki (2000), the International Committee on Harmonization Good Clinical Practices guidelines, and the National Health Service Research Governance Framework. Ethical approval for the study was granted by the National Research Ethics Committee London—Brent, and the Health Research Authority. The study was carried out under a Home Office licence for research with Schedule 1 drugs, and sponsored by Imperial College London.

### Micro-phenomenological interview and analysis

The micro-phenomenological interview method was used to elicit systematic and granular descriptions of the placebo/DMT experience (Petitmengin 2006). The interviewers were instructed to gather a description of the entire experience, but focus on immersion and perceived presences. The interviewee’s memory of the timeline of the experience was supported by reviewing the experience sampling ratings given during the experience. The interview was performed shortly after returning to baseline (normal waking consciousness), and interview questions focused only on the placebo or DMT experience that immediately preceded the interview.

The micro-phenomenological analysis method (Petitmengin et al. 2019, Valenzuela-Moguillansky and Vásquez-Rosati 2019) was used to develop phenomenological categories derived from the interviews in a bottom-up fashion, with categories representing phenomenological distinctions made by participants. The method was then used to assess the order in which categories emerged according to the timeline of each experience, looking for common sequences. This was done by identifying separate phenomenological phases within each experience according to linguistic indicators of a temporal shift, such as ‘after,’ or ‘then,’ and organising the phases and the categories into an individual matrix for each participant. These *phase matrices* were compared using an informal visual search for repeating patterns across participants. Where patterns were observed, an analytical comparison was subsequently performed to quantify its form, variations, and the number of cases in which it occurred.

To assess researcher bias, an independent rater trained in the micro-phenomenological method (author ED) also coded a subset of the data using the primary analyst’s categories, and inter-rater agreement was tested using Cohen’s kappa (Gisev et al. 2013).

Further information on the interview and analysis process is provided at Supplementary material A.

## Results

Micro-phenomenological analysis of the interview data revealed phenomenological structures of immersion and perceived presences, shared across participants. In line with previous research, participants variously described vivid sensory and emotional effects, emergent spatial realms populated with objects and perceived presences, and radical transformations of the usual structure of experience. Precise description of the emergence of these phenomena revealed the numerous dimensions and processes through which immersion occurs. While we took sensory disconnection and interaction or involvement with a world-analogue as a starting point for our exploration, the micro-phenomenological interview and analysis process made clear that these relatively gross forms of immersion are supported by subtler forms, and therefore we present immersion under DMT here as a continuum of subtle to gross phenomenological dimensions. Specifically, while gross forms included relatively complex, gestalt phenomenological configurations like self-location in an emergent environment, and/or engagement in a complex social interaction with a perceived presence, the relatively subtle aspect of immersion includes the engagement of various elemental, experiential faculties that construct and constitute the experience of a world-analogue and a self with and within it, such that an emergent DMT experience could be considered less immersive if it engaged fewer of those faculties. The most obvious examples of such faculties is vision, or audition, however a more comprehensive and nuanced account of these faculties is given in the results below. It is important to clarify that this subtle aspect of immersion should not be conflated with experiential richness (i.e. the amount of contents experienced), but rather relates to the *number* or *range* of *distinct sensory, affective, and cognitive faculties* recruited by an experience as one index of its immersiveness. However, the relationship between subtle and gross immersion is not purely quantitative: gross forms depend not simply on *how many* faculties are engaged, but on *particular combinations* of their engagement and their complex interactions that form coherent gestalts (e.g. a sense of self-location in an emergent environment supervenes on a specific combination of vision, felt space, bodily awareness, and self/world distinction, rather than on the mere sum of independent faculties). Thus, these results set out to illustrate this range of faculties, and the relatively gross dimensions of immersion that they support (i.e. complex gestalts supervening on elemental faculties).

The procedural flow of the analysis started with extracting 125 phenomenological categories, from 23 interviews, representing structural phenomenological distinctions and dimensions that relate to both gross and subtle forms of immersion, and perceived presences. Then, these categories populated a series of 21 phase matrices (two participants excluded due to incomplete interview data; average number of phenomenological phases per matrix: eight, minimum: 5, maximum: 13). Exploratory visual and analytic comparison of the categories and their dynamics in the phase matrices revealed common dynamic patterns across participants.

Because these dynamic patterns emerged on relatively granular dimensions we present them here first (contrary to the procedural flow), followed by a more granular account of the categories and substantiating excerpts from the interviews.

### Dynamics of immersion and perceived presences under DMT

Two of the clearest patterns revealed by analysis of the phase matrices were observed during the initial phases of the experience: (i) structured sequences in early sensory immersion and (ii) structured sequences in the development of spatial complexity and perceived presences; illustrating subtle and gross forms of immersion respectively. Inter-rater agreement on category assignment for these results was ‘almost perfect’ (κ = 0.82), supporting the reliability of the coding process (Gisev et al. 2013).

#### Micro-development of sensory immersion under DMT

**Figure 1:**
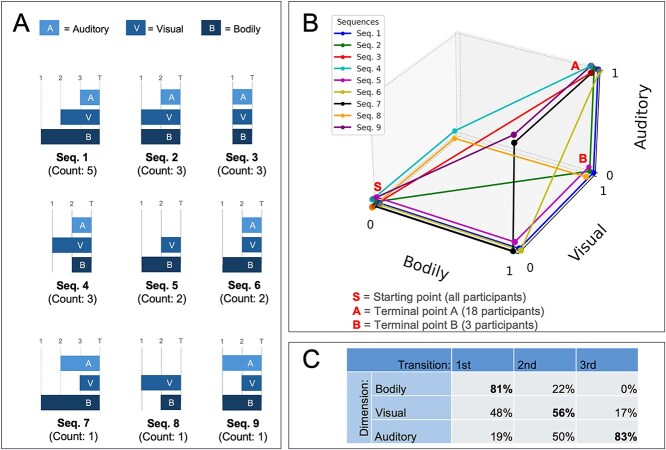
Dynamics of the emergence of sensory immersion under DMT. This figure illustrates typical sequences of emergence for sensory effects across modalities, revealing a hierarchical structure where bodily effects tend to emerge first, visual second, and auditory last. The figure contains: (A) distinct sequences in which sensory effects on the bodily, visual, and auditory modalities were described as emerging, visualised as a series of steps (with variable series length) between first onset (i) and termination (T) of the sequence (i.e. sequence 1: Bodily effects first, visual effects second, and auditory effects third; sequence 3: All emerge at once), and a count of participants to which each sequence applied; (B) all sequences visualised as plot lines through a three dimensional state space, all beginning at point S and terminating at point A or B, with values of 0 and 1 on each axis representing phenomenon absent or present respectively; (C) the percentage of participants for whom a phenomenon emerged at each transition (top percentages in bold; columns do not sum to 100% because multiple categories could emerge at each transition).

Following the systematic micro-phenomenological interview framework, the order in which the sensory modalities were engaged by DMT was relatively precisely described in each interview. Comparing the phase matrices revealed that the order in which phenomena emerged on the visual, bodily, and auditory modalities was variable across participants. This included any non-ordinary sensory effect that was emergent and could be reasonably attributed to DMT, with examples including seeing geometric patterns, feeling ‘pins and needles’ throughout the body, hearing a ringing sound (that was not the scanner). When the emergence of phenomena on these three sensory dimensions was mapped as a series of transitions through a state space, nine distinct trajectories were observed (Fig. 1), with two possible terminal points. Each participant made an average number of 2.85 transitions through the state space (minimum number of transitions:1, maximum: 3). Across these transition sequences a general, hierarchical structure emerged (Fig. 1), whereby bodily effects were most likely to emerge first, visual effects were most likely to emerge second (for sequences that had a second transition), and auditory effects were most likely to emerge third (for sequences that had a third transition). This tendency was strongest for the primacy of bodily effects and the late emergence of auditory effects, while the position of visual effects was more variable.

#### Micro-development of experiences of perceived presences

**Figure 2:**
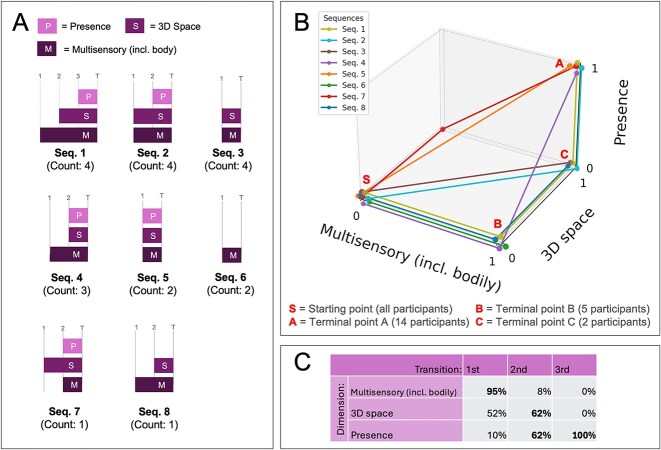
Dynamics of the emergence of perceived presences under DMT. This figure illustrates typical sequences of emergence for multisensory effects, 3D spatial characteristics, and perceived presences, revealing a hierarchical structure where perceived presences tend to only emerge subsequent to multisensory effects and 3D spatial characteristics. The figure contains: (A) distinct sequences in which specific, complex phenomena, each visualised as a series of steps (with variable series length) between first onset (i) and termination (T) of the sequence (i.e. sequence 1: Multisensory (including bodily) first, 3D spatial characteristics second, and presences last; sequence 5: All three emerge at once), and a count of participants to which each sequence applied; B) all sequences visualised as plot lines through a three dimensional state space, all beginning at point S and terminating at point A, B, or C, with values of 0 and 1 on each axis representing phenomenon absent or present respectively; (C) the percentage of participants for whom a category emerged at each transition (top percentages in bold; columns do not sum to 100% because multiple categories could emerge at each transition).

Another structure observed in the phase matrices concerned the development of relatively gross forms of immersion: the emergence of perceived presences in relation to the sensory effects described above, and the emergence of 3D spatial characteristics. In all but one case, 3D spatial characteristics in the emergent DMT experience (i.e. a 3 dimensional spatial relationship between emergent phenomena) were not described as occurring unless bodily effects *and effects in one other sense modality* (i.e. bodily and visual, or bodily and auditory) had previously or concurrently occurred. This combination of sensory effects we refer to hereafter as *multi-sensory effects (including bodily*). Additionally, in all cases where perceived presences were described as emerging (be they seen, felt, heard, or otherwise), this emergence did not occur unless both multisensory effects (including bodily) *and* 3D spatial characteristics had previously or concurrently occurred. When the emergence of these three dimensions was mapped as a series of transitions through a state space, eight distinct trajectories were observed (Fig. 2), with three possible terminal points. Each participant made an average number of 1.8 transitions through the state space (minimum number of transitions:1, maximum: 3). Across these transition sequences a general, hierarchical structure emerged (Fig. 2), whereby multi-sensory effects (including bodily) were most likely to emerge first, 3D spatial characteristics and the perception of presences were equally most likely to emerge at the second transition (for those sequences that had a second transition), and the perception of presences was most likely to emerge in the third transition (for those sequences that had a third transition). These findings highlight the result that perceived presences occur as a part (or as a result) of the immersive landscape comprising multisensory and spatial phenomena.

Results also revealed two distinct types of bodily phenomena contributing to multisensory effects: emergent bodily effects (e.g. vibrations, warm rushes, pressure) experienced by all participants (n = 21), and complete loss of bodily awareness reported by some (n = 11). This loss of bodily awareness could occur before or after the emergence of 3D spatial characteristics, if at all, suggesting that the hierarchical relationship between multisensory effects (including bodily) and 3D spatial characteristics was not dependent on any specific type of bodily experience. Participants could be fully immersed in 3D environments whether they maintained altered bodily awareness or lost it completely.

### Dimensions of immersion and perceived presences under DMT

Now we turn to the fine grained phenomenological categories that the dynamic analysis presented above rested on. Because the concept of immersion under psychedelics is underdefined and underexplored, an intentionally broad purview was taken when abstracting categories, so as to allow both gross and subtle structures of immersion, and perceived presences, to emerge. The categories represent structural phenomenological dimensions, as well as sub-dimensions, and specific distinctions made on these dimensions. Notable categories are described below, supported by excerpts. Categories relating to immersion more broadly are described first, followed by those specific to perceived presences (presences featured in 17 interviews). Category names are presented in small  caps followed by the percentage of interviews a category was identified in (in parentheses). A full list of categories with substantiating excerpts is given at Supplementary material B.

Inter-rater agreement on category assignment was ‘substantial’ (κ = 0.72), supporting the reliability of the coding process (Gisev et al. 2013).

#### Multi-sensory immersion

DMT heavily engaged the modal  senses (100%) in constructing world-analogues. In all interviews, a rich and complex visual (100%) experience was described, which varied on dimensions such as brightness (57%) and colour (100%). Spatial properties of what was seen are detailed below.

auditory (78%) experiences were also described, varying on dimensions of pitch (35%), timbre (65%), and volume (30%). Another dimension of these auditory experiences was musicality (30%), distinguishing between structured music and mere sounds.

While some participants had no  bodily  awareness (48%), others described varied bodily experiences. A recurring tactile (91%) experience was that of pressure on the body emanating from all, or part of, the perceived environment. Notably, tactile feelings persisting from before drug administration (e.g. the feeling of the body against the scanner bed) were at times incorporated into the emergent experience, taking on new significance.

‘I felt I was standing up and dancing. … I was kicking rocks, and I actually felt like I physically hit a rock, … I definitely hit something [lab equipment] that I shouldn’t have.’ (P08).

When not incorporated into the emergent experience, these persistent tactile experiences attenuated the degree of immersion into the experience.

‘I could still feel the sensation of the cannula in my arm, … like a lifeline back to reality.’ (P06).

Thermoreceptive phenomena were also reported (temperature; 70%), at times described as exogenous (e.g. *‘warmth [emanating] from the visuals’*; P08), though at other times described as originating within the body (e.g. a bodily chill).

The distinctness of these visual, auditory, and bodily senses that shaped emergent world-analogues varied. Depending on this degree  of  modal  distinction (48%), they could be described at times as entirely distinct  sensory  registers (35%), while at other times as blended, i.e. synaesthesia (39%). Synaesthetic experiences could involve all of the modal senses, however blends between visual and bodily (tactile, proprioceptive) senses were particularly notable.

“That feeling of electrically vibrating, of being sawn [apart]. … it’s all part of the geometry … the fabric of what you’re feeling. As the picture rips, your body is ripping.” (P18).

In one case, a complete  merge (4%) of the senses was described.

‘It felt like my senses had all conflated ... [into an] amalgamation of everything that I’m sensing … from a sensory point of view, it’s all really merged into one.’ (P06).

#### Semantic complexity

An additional faculty of experience engaged by DMT was semantic  complexity (100%), differentiating, e.g. semantically  simple (91%) visual experiences of shapes and colour, from semantically  complex (16%) visual experiences of recognisable forms fitting categories of objects, architecture, beings, or symbol/language. To clarify this distinction further: semantically simple phenomena represent something relatively limited in scope and well defined in terms of its apparent features (e.g. a triangle, which is defined in terms of its geometry and carries a limited potential for interpreting meaning), while semantically complex phenomena are relatively multi-dimensional and context dependent (e.g. a face, which is defined by a much more complex set of relations above and beyond geometry and has rich potential for interpreting meaning). Phenomena were also at times described as partial representations, suggesting a gradient dimension of semantic complexity.

‘I started thinking about kangaroos… and could kind of see, like, the edges of kangaroos in the blue stuff round the sides… if it would develop more it would have been kangaroos, but it was mainly just blue stuff’ (P08).

Semantic complexity also structured the auditory and bodily aspects of the experience, distinguishing between ‘*sounds*’ (such as a buzzing, or ringing sound) and ‘*sounds of’* recognisable things (such as chanting or laughter); and also distinguishing between abstract bodily feelings (such as a non-specific tingling or vibration) and ‘feelings of’ emergent, perceived objects in the world-analogue. Semantic complexity also structured experiences where bodily sensations were no longer taken as representative of the body, but rather as abstract, dissociated phenomena.

‘I wasn’t really feeling bodily sensations as bodily sensations, or as located in my body. It wouldn’t be quite accurate to say that I felt these sensations as “located in my chest.”’ (P10).

#### Space, seen and felt

The modal sensory experience was complemented by a range of feelings and impressions in faculties outside of the traditional modal senses, here broadly categorized as *amodal*  feelings (65%), at times explicitly identified as a sort of experientially tangible *knowing* that carries a specific form or meaning.

One amodal feeling, key in structuring the experience of emergent spatial environments, was felt  space (43%). The *feeling* of space was described as distinct from, and at times occurring in absence of, any additional modal sensory (visual, auditory) representation of space.

‘When the space opens up to my right, it’s not as though I’m seeing more… it’s this unseeing, felt sense … as though something had been lifted.’ (P07).

Regardless of whether emergent space was seen, heard, or felt, its description varied on spatial  characteristics (100%) such as size (96%), shape (100%), location (100%), density (13%), and dimensionality (96%). The latter distinguished 2d (35%) planar spreads of geometric visuals from 3d (91%) environments. When 3d, these environments had the property of enclosure (43%), whereby the perceived space could be enclosed (30%) or unenclosed (17%). When enclosed, the shape of the enclosure was alternatively described as elementary (13%), e.g. as a simple tunnel or cube, or complex (17%), e.g. as compound elementary shapes forming a series of tunnels or rooms.

‘[The structure I was inside] was huge, like the dimensions of a cathedral… it was flipping over itself … I wasn’t moving ... It’s as if the space itself was moving, a bit like slides, but with a rotating movement… As if the structure was a 3D hexagon, for example… Each side would be a room… A window into a virtual environment.’ (P10).

A fractal-like  recursion (9%) was also possible, whereby the shape of the boundary was arranged in a hierarchical, self-containing, repetition *ad infinitum*. Conversely, unenclosed spatial environments were entirely open and spacious, likened to a garden, or outer space.

The 3D environments described typically followed a hierarchical (52%) figure-ground composition in which figures (52%), including objects, beings, and architecture, inhabited some ground (52%). The objects, architecture, and beings could take 3D forms, however 2D variants within 3D environs were also described. other  dimensions (13%) were described as well, whereby an object or space took on hyper- or otherwise unconventional- spatial dimensions, such as simultaneously revealing the inner and outer perspective of an object.

“[I saw] toys ... with different dimensions folding into each other … as if the side had come away and you could delve through it … it’s not like you’re moving through it, you feel like you’re seeing in more than one dimension” (P06).

Spatial characteristics did not apply only to the sense of an emergent environment, as a number of participants also reported emergent spatial  re-configuration  of  cognitive  phenomena (22%).

‘[There are] shards of memories, or facts… and they all swirl around me… [I am] in this landscape of flying shards, flying in and out of those hexagons connected to those pipes.’ (P11).

#### Felt meanings

Continuing with the amodal feelings engaged by DMT, participants described *feelings* that conveyed complex properties of phenomena above and beyond raw sensory impressions, typically concerning a perceived object’s conceptual and affective relation to the self or other things within the world-analogue. For example, the feeling  of  reality (26%) conveyed a felt sense of the experience (or its contents) being real (22%), or unreal (9%). This feeling of reality was at times described as an overall tone to the experience, while at other times as a gestalt linked to specific sub-properties such as perceived density or clarity (52%) as shown here.

‘That again links to the authenticity of it, the vividness of that feeling. It’s a really strong, crystal clear perception. Even though I guess it’s an illusion, it feels very tangible.’ (P19).

Distinct from ‘real’ was the sense of being ‘unreal,’ described here as appearing both ‘imagined’ and ‘virtual.’

“These faces are happening seemingly in my vivid imagination. There’s this distinction [from parts of the experience that felt ‘real’]. The faces have this virtual quality to them, it’s like they’re not necessarily real” (P03).

The feeling  of  familiarity (65%) conveyed a felt sense of the experience (or its contents) being familiar (61%), or alternatively unfamiliar  or ‘alien’ (13%). Notably, these relational categories were not mutually exclusive.

‘I feel the strange combination of novelty and familiarity… the space looks completely different, but I have this feeling that I’ve been in this place or in this frame of mind before.’ (P11).

#### Immersion and the configuration of self and world

Variation in the delineation and topological arrangement of self and world characterized immersion by governing *how* participants interacted or became involved with emergent world-analogues. This is clear in the synaesthetic bodily experience of the visual landscape described above, however further, higher order categories are described here.

The first of these self/world configuring dimensions is perceptual  position (87%), distinguishing between being within (74%) or outside (57%) an emergent space. A notable recurrent structure was that of looking into a space from outside, described as seeing other worlds through portals or tunnels. A liminal (17%) perceptual position—on the threshold between within and outside a space—was also possible.

‘It’s like a membrane … [It’s] visual and it’s definitely felt… [But] it’s not like I’ve gone all the way through… it’s like I’ve gone into the middle… like I didn’t break through’ (P17).

The next dimension is internalisation (91%), where certain phenomena could be variously described as occurring internal (48%), or external (87%), to the boundary of the body or mind.

‘There are always colours around me, and in me. There were things going through me in that moment.’ (P01).

The experiences also varied on how clear the self/world  distinction (91%) was, meaning that at times a perceived environment was described as clearly  distinct (74%) from the self, while at others the subject described this environment as being indistinct  or  fused (35%) with the self(either fully indistinct/fused, or in the process of becoming indistinct/fused). In some cases this sense of fusion was driven by the synaesthetic merge of the visual and bodily components of the experience, meaning that *feeling* what was *seen* created a sense of *becoming* or *embodying* the environment.

‘It no longer made sense to localise [the vibration previously felt in the body] to the body… [Now, the feeling of vibration] was more closely affiliated with what was going on in my visual field than it was with my body. I became the experience... I actually merged into the scene.’ (P07).

In other cases the usual sensory cues that carved out self and world were lost, such that one’s mind and awareness in a radical state of reconfiguration (beyond any self and world distinction) remained as the main or only describable features.

‘It became an abstract experience. … [Now the experience] wasn’t a thing I was seeing but something that my perception was becoming… It wasn’t a place… [I don’t remember] any visuals or any sensation … [Just a] singularity.’ (P04).

**Figure 3:**
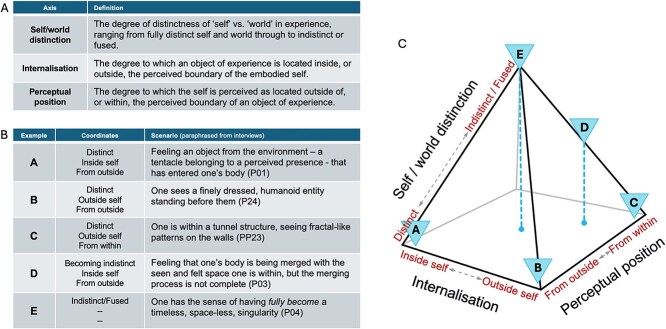
Structures of immersion and self/world configuration under DMT. This figure shows (A) three experiential dimensions of self/world structure defined; (B) non-exhaustive combinations of values (state space coordinates) for these dimensions, with paraphrased examples from interviews; (C) a state space representation of these dimensions, pyramidal in shape due to a non-orthogonal relationship between ‘self/world distinction’ and the other dimensions: When the world or part of it becomes indistinct from the self, then whether the that world/part is within/outside the self (internalisation), and whether the self is within/outside the that world/part (perceptual position), is no longer meaningfully distinguished, thus mediating possibility for one type of gross immersive experience.

Thus, the structural configuration of self and world varied, between moments of the self-perceiving an entirely distinct world (be it a flat image or a fully immersive environment), and moments of self and world becoming fused or indistinct. The fusion of self and world results in the dimensions of internalisation and perceptual position collapsing: the sense of locating the world (or a part of it) as outside or within the self, or locating the self as within or outside a perceived world, no longer apply when self and world are indistinct. This suggests a non-orthogonal relationship between each of these two categories and the category of self/world distinction (Fig. 3), and illustrates a micro-dynamic whereby the dimension of self/world distinction mediates foundational structures of gross immersive experiences of self-location within a world-analogue (e.g. as above when the sense of seeing external environment is undermined when it becomes ‘[no longer a] *thing I was seeing but something that my perception was becoming’*).

Continuing with the self/world configuring dimensions, identification (83%), a relatively subtle immersive dimension, distinguished experiences in which a perceived environment or parts of it represented (or resembled) the self but were not taken literally as part of the self (i.e. not embodied), from those where the environment was experienced as distinctly *not* representing the self.

‘I broke into tears and I saw these sounds, and the meaning of these sounds, expressed visually as a character face of myself, layering into this landscape. … [I saw] exactly the same content and material as my expressions. I was certainly shaping this world.’ (P01).

The previous excerpt also exemplifies a relatively gross immersive dimension that the self/world configuration varied on: interactivity (13%). As in the excerpt, the structure of this interactivity could be indirect (9%), where behaviours had an unintended effect on perceived emergent content. In other cases, a direct (9%) sense of agency permitted the subject to intentionally cause effects on this content through some behaviour. These behaviours could be described as if they were the acts of an agent self in a physical environment.

‘It was a party… [All] around the room, my alien friends… We all had the same cups… someone came to me with this green liquid and they said that this will make you have a good time… I took the green liquid’ (P05).

Or, they could be described as something akin to ‘inner’ acts of mental will, where a desired change is willed to manifest in a perceived landscape without need for any perceived bodily action.

‘I’m no longer aware that my body exists and I need one or I have one… I see this grid of hexagons in light blue, red, yellow and teal. … I can zoom in and out of it and look at this, like you look at 3D models’ (P11).

In most cases no sense of control over the emergent world-analogue was described, outside of attempts to change the receptive  mode (61%) of the self toward it. One basic structure was attention (57%), under which phenomena could be subject  to  attention (43%), or not  subject  to  attention (35%).

‘Even when my attention is going off mind-wandering, the stuff is still there in front of my visual field. … This visual experience has a life of its own. It’s going on whether I’m looking at it or not. I can choose to think about something else and it will still go on doing its thing and then I can choose to pay attention to it.’ (P07).

On the dimension of resistance (83%), emergent phenomena could be accepted  or  allowed (48%)—often phrased as ‘letting go’—or, they could be resisted (35%). We note that *both* accepting and resisting could be seen as ways of interacting or becoming involved with the experience (and thus immersion), and that specific structures of accepting and resisting could incorporate attentional acts—such as resisting by averting attention. However, a full account of these receptive and attentional dynamics was beyond the scope of the interviews.

While descriptions of emergent phenomena dominated the interviews, some participants explicitly described their experience of the dosing environment being completely occluded by the emergent DMT experience, while for others it persisted either through modal sensations or an amodal knowing. This created a gross sense of multiple  contexts (35%) in which the subject described being experientially embedded.

‘I was in this “shades of brown” area, but I was simultaneously aware of the scanner, and the room, all of you … Kind of like a “foot in both worlds”.’ (P04).

#### Phenomena in flux

Immersion varied with the intensifying (52%), dampening (83%), persisting (74%) or ending (65%), of emergent phenomena. The genesis of a phenomenon—its entry into experience—took multiple, structurally distinct forms, referred to here as modes  of  encounter (78%). The first mode of encounter is emergence (87%), under which a phenomenon is experienced as gradually forming within experience. This is distinct from the structure of noticing (13%), under which a phenomenon is experienced as having existed fully formed before coming into experience or attention. Finally, under the structure of revelation (13%), phenomena were experienced as being brought into experience by a perceived presence.

‘One of the figures is presenting something to me… [something] opens up, like a drawer from the wall. In the drawer there’s this object… It’s blue and shaped like a corkscrew… there was a presentation of that object’ (P13).

#### Perceived presences

In addition to revelation, many other categories described the phenomenological structure of emergent perceived presences. For 82% of those experiencing presences, presences were described as *inhabiting* a perceived landscape (i.e. spatially located within a world-analogue), and thus the phenomenological structure of both presences and their environments overlap. Here these structural similarities, as well as key presence-specific categories, are described.

#### The sense of other minds

Presences perceived as inhabiting a particular environment were, like the environment itself, described primarily in terms of modal sensory experiences. Sometimes they were perceived through a single modal sense, while at other times through a combination. When experienced visually, presences were described as discrete objects or entities with a spatial location.

“I’m within this architectural space and I’m seeing these entities… They’re sort of in the periphery of the vision… [They’re] insectoid… They’re long, they are standing upright… [and] made of this tube-like structure… They’ve got heads. I mean, they’ve got, like, these big eyes.” (P17).

When experienced auditorily, presences were heard as chanting, or laughing, or making more abstract sounds. These sounds were at times described as indicating a presence’s location in space.

‘Have you ever run something metal over an electric guitar string and you get a zing sound? Well there were lots of those sounds. … They were happening all around me in space. I interpreted that as there being some small “things” flying around me and making noises.’ (P02).

Presences could also be perceived through the bodily senses.

‘It starts with this presence to my upper right side… There’s this intention towards me that I feel in my body. There’s this pressure happening.’; ‘I feel that these presences are kind of laying me out’ (both quotes: P03).

Presences could also become evident through amodal feeling alone, a phenomenon referred to here as felt PRESENCE (26%). This means that the presence may be entirely unseen, unheard, and untouched, yet a distinct feeling in the subject reveals this presence, at times also communicating its specific characteristics (e.g. gender, spatial form, affect, intentions).

‘I can’t remember seeing a face or thinking that I was ever looking at something that was a “thing”, but there was a deep sense of something being there… It wasn’t there in the way that a normal physical being would be there, it was more of an essence of something that was imbued in the experience that wasn’t physically represented in front of me.’ (P06).

At a single moment, regardless of the dimension of experience through which a presence was sensed, the DMT experience could feature a single presence, or multiple presences, and the presence could be located variously in the compositional hierarchy of a perceived environment, featuring either as the central figure within the scene, or as part(s) of the subordinate ground of the scene.

#### The presence of whom?

**Figure 4:**
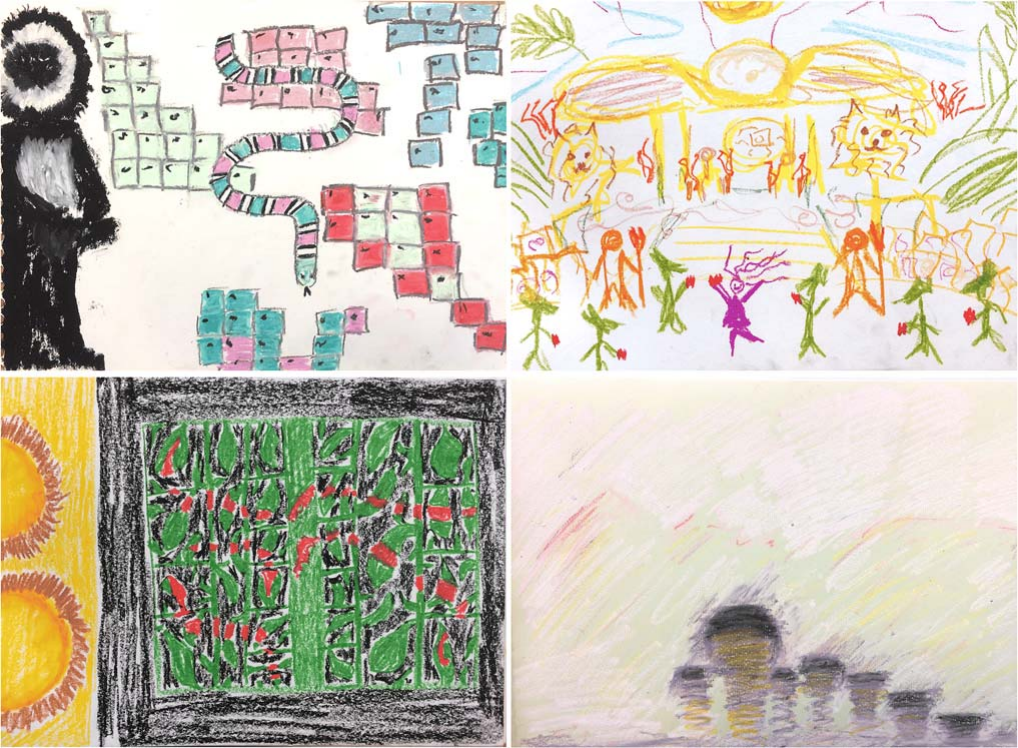
Semantic complexity of perceived presences. Participant illustrations showing variable degrees of semantic complexity in perceived presences: From semantically complex figures (top left: A black and white furred monkey with a colourful cartoon snake; top right: A party of dancing, green aliens holding red cups, in front of a temple, surrounded by plants) to semantically simple and relatively featureless shapes (bottom row). Illustrations were made on the testing day, after the micro-phenomenological interview.

The dimension of semantic complexity was also relevant to perceived presences, which could be relatively experienced as semantically simple or semantically complex (Fig. 4). In regard to perceptions of presences through the modal senses, this distinction between semantically simple and complex is exemplified in the auditory experiences described above of hearing voices or chanting *versus* hearing abstract sounds (e.g. a ‘zing’ sound; P02). It is also exemplified in visual perceptions of presences, where some participants described seeing presences as specific, complex forms.

“I can see the monkey… He is black… He’d be at least 2 stories tall… He’s got a white face, He was a ‘he’... He was on my left side. Like an emperor monkey, you know… they have black, and a white bit, and the fur all grows out.” (P23).

And, in contrasting cases, participants described visual experiences of presences as abstract forms with few features, yet nonetheless sentient and present.

“This space is very colourful … And moving in geometric patterns… And the shapes are also sort of alive. So the shapes are made of some sort of beings. And they’re opening up tunnels for me.” (P14).

In some cases a participant describes feeling complex characteristics about a presence that are more than the sum of its form in the modal senses (referred to by some participants as ‘vibes’ but named more formally here as ambiance, 48%) such as the monkey described by participant 23 above, the experience of which felt *‘fatherly or grandfatherly, that sort of vibe’*. This ambiance suggests a role of amodal feeling even during modal sensory experiences.

More remarkable, however, is the distinction made between semantically simple and semantically complex qualities in perceived presences experienced through amodal feeling alone, without any representation in the modal senses. A presence sensed *via* amodal feelings could be categorized as semantically complex if it possesses relatively complex, specific, identifying characteristics beyond its basic visual or spatial properties.

‘There was this sense the whole time of being taught something and being shown something by a higher power. … [It] has certain feminine qualities to it, which I can’t really understand why. It seems to be a nurturing, teaching type sensation.’ (P06);

In contrast, a presence sensed *via* amodal feelings could be categorized as semantically simple if it had relatively simple characteristics without the same identifying quality, such as basic spatial properties and/or an affective essence at the most.

‘... lots of like...little, kind of, presences… they weren’t like people or animals, they were just like… whisks of alarm, like the emotion was split up into little whisky things… But I don’t think I saw them, I think I just thought like, I can feel them’ (P08).

It is important to reiterate that, despite conveying specific semantic content, these felt characteristics of presences were described as tangible *feelings*, and not as beliefs or rationalisations.

#### Modes of relating

Presences were not merely perceived. Rather, they were often engaged in a variety of complex relationship structures, each with specific roles and various levels of involvement from each party. These social  modes (70%), when engaged (57%) had a direction  of  engagement (43%) that could be passive (57%), meaning the subject was passively being engaged with by the perceived presence, or, active (9%), meaning the subject had an experience of making engaging actions towards the perceived presence.

‘They were winding themselves around my body… half-snake, half-machine... They were coming on to me, to suffocate me and squash my body.’ (P22); ‘I would look more to the monkey for reassurance than the snake’ (P23).

When observing (13%), the perceived presence watched, or examined the subject. Presences were also experienced at times as communicating (13%), whereby an intentional transmission of information from the perceived presences is described, either through sound, visible gesture, or felt meaning. In the case of felt meaning (felt  communication, 17%), this entails an experience of the content of the communication arising within the subject without any clear sensory form—e.g. a felt meaning attributed to a presence but without a heard voice, or a visible gesture or text.

“I’ve got a distinct sense [from the presences] of like, ‘Okay, you’re doing this again’... ‘We were very concerned for you last time. We’re not going to be so concerned for you this time’.” (P17).

When affecting (9%), perceived presences were described as making actions perceived as intentionally trying to elicit an emotional response in the subject, such as taunting or reassuring. When guiding (13%), the perceived presence moved the subject from one spatial location to another. Finally, when manipulating (9%), the perceived presence was experienced as physically manipulating the subject’s body.

‘I feel that these presences are … spreading me in the environment, making my body expand completely in my surroundings… they press and then spread me out in a semi-circular fashion.’ (P03).

Here the receptive modes became relevant too, determining whether interaction with a perceived presence was accepted or resisted, or determining whether a presence was even brought into attention. An additional subject/object configuration of presence experiences was internalisation, under which presences were most commonly described as being located outside of the self-boundary, and in one case described as being located outside of this boundary with a part of the presence entering, across the boundary and into the self. No presences were described as located entirely within the boundary of the self.

“It was large and there were these tentacle-like things coming out of it … and they were entering me.” (P01)

## Discussion

### A dynamic and structured continuum of immersion

This study elucidates structural phenomenological features of experiences of immersion and perceived presences under DMT, demonstrating that even profound and idiosyncratic experiences can be structured and follow shared trajectories fit for scientific study. Using characteristic experiences of sensory disconnection from the environment and interaction or involvement in a world-analogue as a starting point for focusing on immersion, we showed not only core dimensions and structural varieties of these experiences, but also how these relatively gross forms of immersion are supported both structurally and developmentally by subtler forms of immersion, thus proposing experiential immersion under DMT as a complex continuum. This phenomenological characterisation of immersion brings the sub-field in line with other areas, such as the study of virtual reality (VR) technology, which has also yielded complex and multi-dimensional conceptualisations of immersion under VR (Nilsson et al. 2016).

Our dynamic analysis showed that multi-modal sensory phenomena, when not emerging simultaneously, emerge in a reliable sequence of bodily, visual, then auditory, with the primacy of bodily effects and the late emergence of auditory effects being the most robust features of this pattern. This represents a relatively subtle form of immersion whereby an increasing number of distinct sensory faculties are engaged by DMT. We then showed that multisensory effects, including effects in the bodily modality, reliably preceded the emergence of 3D spatial characteristics, which reliably preceded the emergence of perceived presences (again when not emerging simultaneously). This represents experience building towards a gross form of immersion whereby the subject experiences a complex world-analogue, with which they are involved (e.g. spatially located within) and/or interacting. An important next step will be to examine whether these dynamic structures are preserved under a more gradual infusion of DMT (rather than the bolus used here), with the same hierarchy unfolding over a longer timescale.

Through comprehensive micro-phenomenological extraction of categories we also show that subtle immersion can be conceptualized broadly, not just as a recruitment of sensory modalities but rather the number or range of experiential faculties and features that are variably engaged to structure the world-analogue and processes of interaction and involvement in it (e.g. amodal feelings including felt presence, felt space, and feelings of familiarity and reality; see Petitmengin 2007, for a relevant exploration of these dimensions of experience). We propose that while gross forms of immersion supervene on the subtle, they depend not just on the number or range of experiential faculties and features engaged, but on particular combinations of engagement and their complex interactions that form gestalts (e.g. a sense of self location in an emergent geometric landscape populated with objects and beings, which supervenes on a specific range of experiential faculties engaged by DMT, including vision, felt space, *etc.*). This conceptualisation and the dynamic results presented here resonate with research showing that non-pharmacological disruption of multisensory integration can induce both spatial disorientation and presence phenomena (Blanke 2012, Blanke et al. 2014), with bodily perception playing a central role, and again findings in the study of VR, where the interplay between body, self, and perceived environment is linked to the experience of not just other worlds but also other bodies and selves (Bollmer and Suddarth 2022).

Additionally, these findings elucidate how the self/world configuration, including dimensions like self/world distinction, interactivity, internalisation, and receptivity, can govern one’s sense of involvement and interaction with an experience under DMT, and even at times disrupt the sense of self-location in a world-analogue when the self/world distinction collapses. Participants reported alterations in self-world boundaries through multiple pathways, including synaesthetic fusion of tactile and visual modalities, or through the perceiving mind itself becoming the object of experience so as to dissolve the perceiver-perceived distinction. This could be further examined through research with compounds that more reliably alter the relationship between self and world, such as 5-MeO-DMT (Timmermann, Sanders, et al. 2025, Timmermann, Barba, et al. 2025), and during other non-ordinary states which may alter the relationship of self and world during immersive activities requiring expertise (e.g. states of ‘flow’; Csiksentmihalyi 1990). These findings also suggest that micro-phenomenology may be used to more precisely describe experiences of unity (often coarsely conceived as ‘ego dissolution,’ ‘non-duality,’ or ‘mystical experience’; Nour et al. 2016, Stace 1960, Barrett et al. 2015) in a data-driven way, while minimising the imposition of certain cultural and spiritual interpretative confounds (Sanders and Zijlmans 2021).

Broadly, these findings support the view that DMT can render the construction of immersive experience more explicit in awareness, potentially informing the study of immersive experience across other aetiologies, including ordinary experience of the world.

### DMT induced perceived presences

These results highlight the dynamic and multifaceted nature of the perception of emergent presences under DMT. These presences were described as inhabiting an emergent environment or suffusing the broader experience, and were sensed variously through visual, auditory, and tactile perceptions, or in some cases through amodal feelings without clear sensory representation. Presences varied in semantic complexity, from featureless and abstract to identifiable and figurative, and were often experienced as engaging with the participant in different relational modes, such as observing, affecting, or communicating. This confirms the structural dimensions described in previous research without structural focus—e.g. variability in sensory modality, form, and relationship type (Michael et al. 2021, Lawrence et al. 2022), while also expanding and formalising these features as a taxonomy of structural dimensions to support future comparative phenomenological research. Presence phenomena are not unique to DMT and have been described in neurological disorder, extreme physiological stress, and intentionally induced illusion (reviewed in Alderson-Day et al. 2023). Comparative neurophenomenological research could identify etiology-specific characteristics of these phenomena, both phenomenological and neurobiological.

That presences represented the ‘peak’ of the dynamic structures we observed reveals that these high-order phenomena may also depend on the integration of more fundamental elements, namely multi-sensory effects on the bodily and other sensory modalities, and the subsequent (or concurrent) emergence of 3D spatial characteristics (*cf.*  Gómez-Emilsson 2016, for previous observations on hierarchical structure of DMT experience). Hierarchy amongst immersive qualities was not ubiquitous, however, because semantic complexity of the visual sensory experience (i.e. whether the subject had simple *versus* complex visual hallucinations; Shenyan et al. 2024) did not emerge as a predictor of perceived presences. This suggests that the hierarchical dependencies identified here may be specific to faculties involved in constructing the spatial and sensory scaffolding of immersive experience, rather than those involved in the interpretive elaboration of its contents. Whether semantic complexity operates on a different developmental timescale, or follows a different kind of dependency structure, warrants further investigation, as does the micro-development of presences in experience (i.e. more narrow-focused interviewing on moments of presence formation and recognition).

It is notable also that while we observe presences at the peak of a hierarchy of emergent effects under DMT, under Parkinson’s disease symptoms of presence phenomena often developmentally precede those of visual hallucinations (Fénelon et al. 2000), suggesting multiple pathways to presence experiences and underscoring need for further study.

### Implications and neurophenomenology

The present findings have implications beyond the descriptive characterisation of DMT experiences. By pointing to structured dependencies in how immersion and perceived presences under DMT unfold over time, this study shifts the explanatory focus from isolated experiential contents to the processes through which they develop. This process-oriented account places meaningful constraints on future neurophenomenological models of psychedelic experience. At the same time, the phenomenological dimensions identified here provide a structured basis for integration with neurophysiological data, delineating the experiential phenomena that any neurobiological account would need to explain (Varela 1996, see Timmermann et al. 2019 for an example of DMT neurophenomenology). Neural explanations must be capable of accounting not only for the presence or absence of specific experiential features, but also for their acute phenomenological development (Timmermann, Bauer, et al. 2023). As a specific example, the finding that perceived presences typically emerge only once multisensory and three-dimensional spatial structures are established suggests that neural accounts which privilege social or agentive processing in isolation are likely to be incomplete.

More broadly, this study demonstrates how micro-phenomenology, a relatively precise and formal method that privileges phenomenological structure and dynamics, can support the scientific study of consciousness. By combining systematic and rigorous micro-phenomenological interview processes, fine-grained category extraction, and temporally sensitive dynamic analysis, the approach offers a replicable template for studying other non-ordinary or altered states of consciousness, e.g. building on recent studies of mind-wandering, meditation, micro-dreams, and dreamless sleep (Petitmengin et al. 2017, Nave et al. 2021, Yang et al. 2024, Nielsen 2017, Alcaraz-Sánchez 2023).

This work also can facilitate further studies of psychedelic immersion across compounds, using the phenomenological specificity given here to probe not just the *depth* of immersion under each compound, but also the *types* of immersion. However, the present account of immersion and perceived presences should not be considered complete, and so the same methodological template should be used for further work refining the construct of immersion under psychedelics, performing more targeted interviews on aspects of immersion that are currently underdeveloped here, such as the experience of being embedded in multiple experiential contexts, the feeling of (un)reality, or the act of resisting *versus* allowing experience.

### Limitations

Firstly, in regard to limitations, the lab setting, experience sampling procedures, and concurrent fMRI scanning may have influenced the phenomenology (Hartogsohn 2017, Roseman et al. 2024), though the described experiences aligned with reports from both naturalistic and other lab contexts (Strassman 1996, Michael et al. 2021, 2023, Lawrence et al. 2022).

Additionally, while micro-phenomenology aims to mitigate reporting biases such as selective recall and post-hoc rationalisation, we cannot be certain that they are eliminated entirely. This highlights a need for validating these phenomenological results *via* constraints stemming from neurobiology (Varela 1996).

## Conclusion

This study reveals how experiences of immersion and perceived presences under DMT are structured and unfold over time, while demonstrating the viability of micro-phenomenology for mapping non-ordinary states. The findings move beyond traditional conceptual and methodological constraints to offer a granular and temporally sensitive phenomenological account, showing that immersion is a multidimensional, processual configuration of experience, and that perceived presences typically arise within this broader immersive structure rather than in isolation. This opens new ground for comparative studies of altered consciousness, as well as more precise neurophenomenological analysis. In doing so, it positions both DMT and micro-phenomenology as powerful tools for advancing psychedelic science and the broader study of consciousness.

## Supplementary Material

Sanders_Revised_Clean_Supplementary_DMTMicrophenomenology_copy_niag015

## Data Availability

Data from this manuscript is available upon reasonable request to the corresponding authors.
