# Supplementary material for: Micro-phenomenology of immersion and perceived presences under DMT
Source: Neurosci Conscious. 2026 Jul 14;2026(1):niag015. doi: 10.1093/nc/niag015 (PMC13366535; doi:10.1093/nc/niag015)
Supplement: Sanders_Revised_Clean_Supplementary_DMTMicrophenomenology_copy_niag015 [file sanders_revised_clean_supplementary_dmtmicrophenomenology_copy_niag015.docx]

## 8. Supplementary material

### Supplementary material A: Micro-phenomenological methods

##### **Additional information on interview method**

Per the specifics of the method (Petitmengin, 2006), the interviewer guided the participant to immerse themselves in the memory of the experience and describe it on both the synchronic (within-moment) and diachronic (across-moment) dimensions. After establishing a rough overview of the experience, the interviewer revisited key moments of the experience with the participant, using an iterative process of refining questions to encourage more granular and specific descriptions of this phenomenal character. The questions asked were ‘content free’ so as to avoid inducing false recall and confabulation (example: when you felt X, what is it that you felt?; or, when X happened, how did it begin?). When choosing moments/aspects to refine, the interviewers prioritised those relevant to immersion and perceived presences. Additionally, the interviewer frequently used the participant’s words to repeat parts of the description back to them, to check for any misunderstandings. Where content that did not concern the acute phenomenal character of the experience came up, such as generalisations or judgements about the experience, the interviewer reoriented the participant back to describing the qualities of the experience itself.

The timeline of the experience explored in the interview started from the moment just before drug administration and finished at the perceived end of the subjective effects, or the moment of exiting the scanner if that came first.

To establish the timing of the experience the interviewer referred to the experience sampling ratings given during the acute experience throughout the interview, so that participants could connect specific phenomena to specific ratings and their corresponding time points. Interviews were performed in a separate room within the facility, nearby to the scanning room, and were audio-recorded.

##### **Additional information on analysis method**

The analysis had three distinct stages: 1) a pre-processing phase, 2) a category extraction phase, and 3) a category dynamic analysis phase.

For the purpose of clear communication the analysis is described here in a linear fashion, however in practice (and by design) the flow of the analysis is iterative, with results of later stages at times triggering re-interpretation of earlier stages as the phenomenological structures gradually emerge.

##### ***Pre-processing:***

Once the audio recorded interviews were transcribed, text that did not concern the acute subjective character of the experience (the satellite dimensions of a description; Petitmengin, 2019) was excluded. The remaining text was re-ordered according to the timeline of the experience.

##### ***Category extraction:***

The transcript of each interviewee's words was broken down into sub-sections (*descriptemes*; Petitmengin, 2019) and assigned a category according to the phenomenological structure to which the descripteme referred. The length of each descripteme was determined by how much text was necessary to convey the applied category, ranging from a few words to a few sentences. In many cases multiple categories applied to a single descripteme. Once these descriptemes and categories were identified across the dataset they were compared and categories deemed to be describing the same phenomenon were merged, thus grouping similar descriptemes. Categories that described distinct but related phenomena were grouped, and superordinate categories that described this grouping were abstracted, thus forming hierarchical categories which represent the common phenomenological distinctions made by participants in their descriptions. This hierarchy of categories is presented in a matrix, with examples from the text, at Supplementary Material B.

##### ***Category dynamics:***

Each interview was segmented into phenomenological phases in the timeline of the experience, using linguistic indicators of temporal shifts (e.g., utterances like ‘then’, ‘after that’, or ‘suddenly’ would trigger a new phase). Thus, a phase contains descriptions of concurrent phenomena, and a subsequent phase indicates new phenomena emerging, or existing phenomena ending or otherwise transforming. This sequence of phases was unique for each participant, and the minutes at which each phase started and ended per the interview text was noted, or inferred from quantitative data where not described.

Following the micro-phenomenological analysis method of *dynamic lines* (Valenzuela-Moguillansky, 2019) categories and phases were organised into a matrix, representing which categories were active in each phase and how this changed from phase to phase for each participant. If a phase was not distinguishable from its preceding or following phase on at least one of the chosen phenomenological dimensions, then the identical phases were merged. These *phase matrices* were compared using an informal visual search for repeating patterns. Where such patterns were observed, an analytical comparison was subsequently performed to quantify its form, variations, and the number of cases in which it occurred.

##### ***Inter-rater agreement:***

To assess the reliability of the coding process, a test of inter-rater agreement was conducted using Cohen’s kappa (Gisev et al., 2013). This was applied to both sections of the results: (1) the structural categories describing the various dimensions organizing the experiential landscape, and (2) the dynamic categories characterizing the unfolding of that landscape over time.

For the structural categories (1), a subset of eight participants was selected using a random number generator. Given the large number of categories, they were grouped into clusters (modal senses, amodal feelings, self/world configuration, and transmodal properties). A second round of random selection was then used to assign each participant a specific phase and a category cluster. The primary analyst and an independent rater each coded the selected phases using the selected category clusters in each transcript. The coding of each analyst was compared, and agreement on the presence or absence of each category within the selected phases was measured using Cohen’s kappa, averaged across participants. Due to a substantial level of agreement from this first round (κ=0.72), an adjudication process to resolve disagreements was not performed.

For the dynamic categories (2), the same eight participants were used. Both raters reviewed the transcripts for each of these participants and assigned a sequence for each (one sequence for bodily, visual, auditory, and another for multisensory, 3D, and perceived presence), and inter-rater agreement on the sequences assigned was again measured using Cohen’s kappa. A small number of disagreements emerged, and some were resolved through an adjudication process between the raters (first round κ=0.78 and 0.35; second round κ=0.81 and 0.83; in regard to the shift from 0.35 to 0.83 on the second kappa value, it is worth noting that this was due to an misaligned understanding of a category definition between raters in the first round, and a common definition was reached in the adjudication process).

### Supplementary material B: Full list of categories

This supplement shows the full list of categories relating to immersion under DMT. The categories are organised hierarchically. These hierarchical relationships are indicated by arrows (each arrow concerns the category that is to the left, and above), according to the formalisms of the micro-phenomenological method (right angled arrows represent the operation of aggregation/fragmentation, connecting categories to their *composite* sub-categories; diagonal angles represent the operation of generalisation/specialisation, connecting categories to specific *sub-types*; Petitmengin, 2019; Valenzuela-Moguillanksy, 2019). Descriptions and examples of each category are given, along with frequencies, which indicate the number of interviews in which a category is grounded (and not necessarily the number of experiences that a phenomenon featured in, due to the possibility that some details of experience were not described).

| **Category** |  |  |  |  |  | **Freq.** | **Description** | **Example** |
| --- | --- | --- | --- | --- | --- | --- | --- | --- |
| **Immersion under DMT** |  | | | | | | | |
| Senses & feelings |  |  |  |  |  | 23 | Senses and feelings forming components of the landscape of DMT experience | It was just perfect ... The symmetry of it and the precise detail of it, and the colours of it, and the crystal clarity of it. (P19) |
|  | ↳Modal senses |  |  |  |  | 23 | Specific sense modalities on which phenomena are described as occuring | (See bodily, visual, auditory) |
|  |  | ↳Degree of modal distinction |  |  |  | 11 | The degree to which the modal senses are experienced as distinct sensory registers | (See distinct sensory registers, synaesthesia, complete merge) |
|  |  |  | ↖Distinct sensory registers |  |  | 8 | A specific value of degree of modal distinction under which the senses are experienced as distinct sensory registers | There was no connection between the audio and the visuals anymore. (P09) |
|  |  |  | ↖Synaesthesia |  |  | 9 | A specific value of degree of modal distinction under which the distinction between sensory registers is less clear | It’s happening in my visual field, but is also happening in the whole body. My whole body is vibrating with these pin pricks of electricity… and its like a synaesthesia between the visual scene and the body and the physiology and the music.(P18) |
|  |  |  | ↖Complete merge |  |  | 1 | A specific value of degree of modal distinction under which the distinction between sensory registers is lost | It felt like my senses had all conflated into one (P06) |
|  |  | ↳Visual |  |  |  | 23 | The sensory register that is seen | (See brightness, colour, shade, vibrance) |
|  |  |  | ↳Brightness |  |  | 13 | The degree of lightness/darkness to a visual phenomenon | There was in the right periphery a bright light, as if there was another scene waiting somewhere back there (P13) |
|  |  |  | ↳Colour |  |  | 23 | The colour of a visual phenomenon | (See shade, saturation, polychromatic, monochromatic) |
|  |  |  |  | ↳Shade |  | 22 | The particular shade of a colour | It was all rippling with green and red (P02) |
|  |  |  |  | ↳Vibrance |  | 3 | The vibrance of a colour, which may encompass both saturation and brightness | The colors are again very bright and vibrant still in this blue yellow teal red spectrum (P11) |
|  |  | ↳Auditory |  |  |  | 18 | The sensory register that is heard | (See volume, pitch, timbre, musicality) |
|  |  |  | ↳Volume |  |  | 7 | The relative loudness of a heard phenomenon | the music had started to fade a little bit. It was still coming but it wasn’t as loud and as fast or as intense as it had been (P12) |
|  |  |  | ↳Pitch |  |  | 8 | The relative pitch of a heard phenomenon | During the early part of the experience it did sound like there was some kind of “gwhooo”. I don’t know if it’s a vibration or a droning sound ...  It wasn’t particularly high-pitched (P19) |
|  |  |  | ↳Timbre |  |  | 15 | The distinctive character of a heard phenomenon, unrelated to pitch or volume | I noticed that the beating sound of the scanner ... was like tribal drumming and chanting... It was switching between drumming and chanting (P02) |
|  |  |  | ↳Musicality |  |  | 7 | The experience of heard phenomena as music, in a relational structure with other sounds | I feel like I’m hearing notes that could be part of a melody, and then it kind of goes away. [It] is going in and coming out. I feel like there is a melody that was played. (P25) |
|  |  | ↳Bodily |  |  |  | 23 | The cluster of sensory registers experienced as through the body | (See tactile, position, temperature) |
|  |  |  | ↖Tactile |  |  | 21 | A felt sense of contact between the body and another object or itself | I did feel a pressure of something hitting me (P12) |
|  |  |  | ↖Position |  |  | 6 | A felt sense of the position of the body and its constituent parts in space | I feel like the perspective that I’m facing changes and it’s like my head is now in a sitting down position, it’s not my whole body that’s in a sitting down position, just my head. (P03) |
|  |  |  | ↖Temperature |  |  | 16 | A felt sense of temperature | the warmth is encompassing the whole of my body, not just some parts of it, so my whole body is being governed by this experience and by this space (P03) |
|  | ↳Amodal feelings |  |  |  |  | 15 | Felt senses that defy categorisation into modal senses | (See felt space, temporality, etc.) |
|  |  |  | ↖No bodily awareness |  |  | 11 | Experiencing an absence of all bodily sensations | My body wasn’t there, I definitely had no sensation of being anything other than my mind. (P06) |
|  |  | ↳Felt space |  |  |  | 10 | A felt sense of the spatial characteristics of an inhabited environment | I had a very clear sensation, or a sense that the right side of my world opened up. ... It felt like there was a sort of boundary which had opened up. (P07) |
|  |  | ↳Temporality |  |  |  | 16 | A felt sense of the qualities of time passing | (See a sense of time, no sense of time) |
|  |  |  | ↖Temporal |  |  | 16 | A felt sense that time is passing | I think [the movement of the rooms was] pretty quick. I’d say a few seconds in each room (P10) |
|  |  |  | ↖Atemporal |  |  | 4 | A felt sense that time is not passing, or that the dimension of 'time' is irrelevant or otherwise not present in the experience | There was no time, or time was infinite maybe (P14) |
|  |  | ↳Feeling of reality |  |  |  | 6 | A felt sense of whether or not phenomena are part of, or correspond to, 'reality' | (See real, unreal) |
|  |  |  | ↖Real |  |  | 5 | A felt sense that phenomena are part of, or correspond to, 'reality' | The ambience is a feeling of importance. It feels extremely important and powerful and very real. It feels very very real. (P03) |
|  |  |  | ↖Unreal |  |  | 2 | A felt sense that phenomena are not part of, or do not correspond to, 'reality' | The faces have this virtual quality to them, it’s like they’re not necessarily real and that’s why they also seemed quite harmless maybe (P03) |
|  |  | ↳Felt presence |  |  |  | 6 | A felt sense that a sentient other is present, which may or may not be accompanied by any kind of representation of that presence in the modal senses | I can’t remember seeing a face or thinking that I was ever looking at something that was a thing, but there was a deep sense of something being there. If something was there, it wasn’t there in the way that a normal physical being would be there, it was more of an essence of something that was imbued in the experience that wasn’t physically represented in front of me. (P06) |
|  |  | ↳Felt communication |  |  |  | 4 | An experience of some communication content that is felt, rather than heard or read, and attributed to a felt presence | It almost felt like the entities, when they were doing that, it was almost like ‘this is what you need to do to be in this place’ sort of feeling. ... This was their communication feeling. (P03) |
|  |  | ↳Ambiance |  |  |  | 11 | A feeling of some complex characteristic (beyond basic sensory or visual/spatial qualities), experienced as emanating from a perceived environment or its contents | There’s always this atmosphere or this ambience ... The ambience is a feeling of importance. (P03) |
|  |  | ↳Feeling of familiarity |  |  |  | 15 | A felt sense discerning whether an object of experience is familiar or unfamiliar - particularly notable because unrecognised objects can feel familiar | (See familiar, alien / novel) |
|  |  |  | ↖Familiar |  |  | 14 | A felt sense of familiarity with specific phenomena, with or without a specific memory of the previous encounter | I feel a feeling of return, familiarity, this feeling of ‘I’ve been here before’. Nothing like ‘oh I had forgotten’ or anything, or maybe a bit, but this beginning of a sense of familiarity, like you’re walking back to your childhood home or something like that. (P03) |
|  |  |  | ↖Alien / novel |  |  | 3 | A felt sense of unfamiliarity with specific phenomena | It really feels like what I’m seeing hasn’t come from my own head… Because I’m just so surprised by it, it’s so otherworldly. (P06) |
|  | ↳Transmodal properties |  |  |  |  | 23 | Dimensions applicable to two or more modal and/or amodal senses and feelings | (See clarity, semantic complexity, etc.) |
|  |  | ↳Clarity |  |  |  | 12 | The degree to which certain phenomena within an experience are more or less defined, distinct, or coherent, or intelligible than others. | It was just perfect ... The symmetry of it and the precise detail of it, and the colours of it, and the crystal clarity of it. (P19) |
|  |  |  | ↖Clear |  |  | 6 | A specific degree of clarity under which phenomena are described as clear, sharp, etc. | That again links to the authenticity of it, the vividness of that feeling. It’s a really strong, crystal clear perception. Even though I guess it’s an illusion, it feels very tangible. (P19) |
|  |  |  | ↖Unclear |  |  | 6 | A specific degree of clarity under which phenomena are described as unclear, blurred, etc. | It has a spatial configuration but doesn’t have a clear image (P03) |
|  |  | ↳Semantic complexity |  |  |  | 23 | The degree to which a phenomenon represents something limited and well-defined in terms of its apparent properties (e.g.: a triangle defined in terms of its geometric properties) versus something multi-dimensional and context-dependent (e.g.: a face defined in terms of relatively complex relations between elements and gestalt properties). | (See semantically simple, semantically complex) |
|  |  |  | ↖Semantically simple |  |  | 21 | A specific degree of semantic complexity, at which phenomena are perceived in terms of elementary features, such as shapes, colours, etc., without being a part of more complex gestalts such as objects, beings, or architecture. | As it continued to die down, the structures rather than being really tight and compact, the shapes were still there but they were bigger as though they were fading but they were bigger so you still have like a cube or a square or a triangle or a pyramid, but actually the edges it seemed bigger and the shapes seemed to be floating away. (P12) |
|  |  |  | ↖Semantically complex |  |  | 16 | A specific degree of semantic complexity, at which phenomena are perceived in terms of complex gestalts, such as objects, beings, or architecture. | (See objects, architecture, beings) |
|  |  |  |  | ↖Objects |  | 4 | A specific value of semantically complex, under which there is a sensory representation of objects (familiar and recognisable or unrecognisable) | The temple was still mustard yellow, the plants were huge and bright green. I can’t remember whether the aliens were yellow or green. ... Everyone was talking and dancing, having a good time ... it was like one of those American parties where everyone has the same coloured cup. ... Someone came to me with this green liquid and they said that this will make you have a good time. (P05) |
|  |  |  |  | ↖Architecture |  | 10 | A specific value of semantically complex, under which there is a visual representation of architecture | The temple was still mustard yellow, the plants were huge and bright green. I can’t remember whether the aliens were yellow or green. ... Everyone was talking and dancing, having a good time ... it was like one of those American parties where everyone has the same coloured cup. ... Someone came to me with this green liquid and they said that this will make you have a good time. (P05) |
|  |  |  |  | ↖Beings |  | 14 | A specific value of semantically complex, under which there is a visual representation of beings | [They look like] a kind of Buddhist god... Human I think. Human, in nice religious-type clothing. It wasn’t gold and purple, but this sort of regal feel... Like fancy clothing. (P24) |
|  |  |  |  | ↖Symbol / language |  | 3 | A specific value of semantically complex, under which there is a visual representation of language (the meaning of the language can be known or unknown) | I start to see the language. ... This language is all stacked up in these tube-like structures. (P17) |
|  |  | ↳Spatial characteristics |  |  |  | 23 | Dimensions that organise the sense of space, irrespective of the sense modality or amodal sense, through which it is experienced | (See dimensionality, size, shape, etc.) |
|  |  |  | ↳Dimensionality |  |  | 22 | The number of spatial dimensions in which a phenomenon is represented | (See 2D, 3D, other dimensions) |
|  |  |  |  | ↖2D |  | 8 | A specific degree of dimensionality, with phenomena represented in 2 spatial dimensions | It was this beautiful image, like a curtain... just geometrical patterns...They have sharp edges, they’re full of colour, yellow and orange and red and all warm colours, all warm and very bright colours that interchange themselves... They remind me of origami, but 2D origami. (P05) |
|  |  |  |  | ↖3D |  | 21 | A specific degree of dimensionality, with phenomena represented in 3 spatial dimensions | The blackness of the visual field became a depth, like a tunnel and then the crinkly shape took more of a radial symmetry. I remember thinking that this seemed like something I was moving towards, and the centre of it becoming increasingly intricate in patterns. I felt as if I was going through something. (P07) |
|  |  |  |  | ↖Other dimensions |  | 3 | A specific degree of dimensionality, with phenomena represented in dimensions above or beyond the third dimension | toys ... with different dimensions folding into each other ... like when you see on YouTube how a combustion engine works and it looks inside it and you see the pistons going up as if the side had come away and you could delve through it. The way that the cubes were morphing and turning in on themselves is a bit similar to that but it’s not like you’re moving through it, you feel like you’re seeing in more than one dimension, (P06) |
|  |  |  | ↳Size |  |  | 22 | The degree to which certain phenomena are relatively large or small | it feels like a rectangular room or hall, or big, cavernous, space (P06) |
|  |  |  | ↳Shape |  |  | 23 | The perceived shape of an object of experience | I was watching the stripes… [and] the folding of the stripes. And the different shapes that were collapsing on those hexagons of different colors, and kaleidoscope with other visuals (P11) |
|  |  |  | ↳Density |  |  | 3 | A felt sense of the density of seen phenomena or felt space | I saw what looked like buildings. ... But very angular, very geometric. (P12) |
|  |  |  | ↳Location |  |  | 23 | The direction and distance of a phenomenon relative to the observer or other phenomena | It felt like it was expanding and increasing in density (P07) |
|  |  |  |  | ↖Close |  | 19 | A specific value of location, whereby a phenomenon is experienced as located relatively close to the self in space | It felt as if I had this structure above me ... It was not so clear-cut, but the ceiling itself was, on the scale, comparable to cathedrals. 25, 30 metres. (P10) |
|  |  |  |  | ↖Distant |  | 8 | A specific value of location, whereby a phenomenon is experienced as located relatively far from the self in space | It was as if it was another layer where all of this stuff happened and there was nothingness above me and below me (P01) |
|  |  |  | ↳Enclosure |  |  | 10 | The property of an inhabited or otherwise perceived space (not) having an enclosing boundary | (See enclosed, unenclosed) |
|  |  |  |  | ↖Unenclosed |  | 4 | A specific value of enclosure, with the inhabited or otherwise perceived space not having an enclosing boundary | [The pattern] expanded to fill my entire perception, and felt like I was existing in a vast continuous space. A continuous, empty plane (P04) |
|  |  |  |  | ↖Enclosed |  | 7 | A specific value of enclosure, with the inhabited or otherwise perceived space having an enclosing boundary | There were geometric patterns, but they weren’t in the front like the previous ones, they were on the sides, and they were more like an igloo around me. (P05) |
|  |  |  |  |  | ↖Elementary | 3 | A specific value of enclosure, under which the shape of the enclosing boundary is elementary, such as a tunnel or rectangular room | There were geometric patterns, but they weren’t in the front like the previous ones, they were on the sides, and they were more like an igloo around me. (P05) |
|  |  |  |  |  | ↖Complex | 4 | A specific value of enclosure under which the shape is a complex combination of shapes/areas with internal boundaries and openings, such as a hallway with adjoining rooms, or a network of interconnected tunnels | I see this grid of hexagons in blue and light blue. Red, yellow and teal. Those hexagons- again they are, like, giant height. I can zoom in and out of it and look at this like you look at 3D models, from all directions, also from the inside, and inside of each of those hexagons are again, like, pipes connecting them (P11) |
|  |  |  | ↳Composition |  |  | 12 | The specific form of spatial relation between phenomena as they cohere into perceived scenes or environments | (See hierarchical, fractal-like) |
|  |  |  |  | ↖Hierarchical |  | 12 | A specific form of composition, under which certain phenomena form the 'ground', or overall space, in which other phenomena are contained and featured more prominently as 'figures' | (See figure, ground) |
|  |  |  |  |  | ↳Figure | 12 | A specific component of hierarchy; the content featured in the ground | So I’m seeing through this rectangular room with pink and green tiles on the floor... in the center I might see one of those toys (P06) |
|  |  |  |  |  | ↳Ground | 12 | A specific component of hierarchy; the ground in which content is featured | So I’m seeing through this rectangular room with pink and green tiles on the floor... in the center I might see one of those toys (P06) |
|  |  |  |  | ↖Fractal-like |  |  | A specific form of composition in which a compositional element recursively contains itself ad infinitum | They were like really big sheets of, kind of, digital depictions of material, and they would shimmer ... they had squares within squares, and, like, stretched like as far as you can see, and then beyond. (P08) |
|  |  |  | ↳Multiplicity |  |  | 23 | The number of times a phenomenon is concurrently instantiated in an experience | (See multiple, single) |
|  |  |  |  | ↖Multiple |  | 21 | A specific value of multiplicity, under which a phenomenon is instantiated multiple times, concurrently, within an experience | the space [is] filled with these tunnels and shapes (P14) |
|  |  |  |  | ↖Single |  | 12 | A specific value of multiplicity, under which a phenomenon is instantiated only once, concurrently, within an experience | So I’m seeing through this rectangular room with pink and green tiles on the floor... in the center I might see one of those toys (P06) |
|  |  | ↳Dynamic characteristics |  |  |  | 23 | Dimensions that organise and characterise the temporal unfolding of an experience | (See speed, movement, etc) |
|  |  |  | ↳Speed |  |  | 21 | The temporal rate at which a phenomenon is undergoing a certain change or process | There was this sense of constant movement. And fast. (P10) |
|  |  |  | ↳Movement |  |  | 20 | The continuous movement of a phenomenon from one spatial location to another | (See moving, stationary) |
|  |  |  |  | ↖Moving |  | 19 | An experience of a phenomenon that is moving from one spatial location to another | There was this sense of constant movement. And fast. (P10) |
|  |  |  |  | ↖Stationary |  | 6 | An experience of a phenomenon that is static in its spatial location | It’s as if I wasn’t moving (P10) |
|  |  |  | ↳Mode of encounter |  |  | 18 | The process through which a phenomenon comes into experience | (See emergence, noticing, revelation) |
|  |  |  |  | ↖Emergence |  | 20 | A specific mode of encounter, under which the perceived process of a phenomenon taking form is experienced either in the centre or periphery of attention | the blackness of the visual field began to take on some structure and began to be almost crinkled like black paper with creases. So it looked more three-dimensional, it was mainly lines and it had symmetry down the vertical midline. It was opening up and it was the shape of a teardrop or an ellipse along the long axis, top to bottom. As time was going and the seconds were passing it was becoming increasingly more intricate (P07) |
|  |  |  |  | ↖Noticing |  | 3 | A specific mode of encounter, under which the perceived process of a phenomenon taking form is not experienced at all, with the phenomenon entering attention perceived as fully formed | I noticed there was a room, a panel of yellow which seemed like a room. (P02) |
|  |  |  |  | ↖Revelation |  | 3 | A specific mode of encounter, under which a phenomenon perceived as fully formed is experienced as being revealed and brought into experience by some perceived external agent | The shapes are made of some sort of beings. And they're opening up tunnels for me. (P14) |
|  |  |  | ↖Persisting |  |  | 17 | The experience of a phenomenon persisting from previous moments of experience | Even when my attention is going off mind-wandering, the stuff is still there in front of my visual field ... It's going on whether I'm looking at it or not. I can chose to think about something else and it will still go on (P07) |
|  |  |  | ↖Ending |  |  | 15 | The experience of a phenomenon ceasing | I could feel the being still with me. And then I could not feel him anymore. (P24) |
|  |  |  | ↳Frequency / rhythm |  |  | 6 | The specific frequency with which a phenomenon comes in and out of experience over time | It was green and then it was red electrical pulses [rippling through]. ...  The rippling was regular but in waves. (P02) |
|  |  |  | ↳Trajectory |  |  | 20 | The dynamic path on which a specific phenomenon is changing by some degree, in some direction, on some dimension over time (e.g.: a sound increasing in volume) | Yeah, so it's just fading. I mean, it's very subtle. Same stuff, but just no real new stuff happening, but just a fading of that stuff. And that just means that somebody's turning down a dimmer switch. So all that's still there is just the light, gradually, the intensity of light fading. (P17) |
|  |  |  | ↖Intensifying |  |  | 12 | A perceived phenomenological trajectory on which a quality of a certain phenomenon is experienced as increasing | Yes [the patterns were coming at me]. Increasing speed. It started, and then moved past me and then around me. (P24) |
|  |  |  | ↖Dampening |  |  | 19 | A perceived phenomenological trajectory on which a quality of a certain phenomenon is experienced as decreasing | Yeah, so it's just fading. I mean, it's very subtle. Same stuff, but just no real new stuff happening, but just a fading of that stuff. And that just means that somebody's turning down a dimmer switch. So all that's still there is just the light, gradually, the intensity of light fading. (P17) |
| Self/world configuration |  |  |  |  |  | 17 | The dimensions that structure the perceived boundary between self and other | (See perceptual position, internalisation, etc.) |
|  | ↳Perceptual position |  |  |  |  | 20 | The perceived locus of sense perception, situated at a particular point in space | (See within, outside, liminal) |
|  |  | ↖Within |  |  |  | 17 | A specific value of perceptual position, under which the locus of sense perception is perceived as within, and surrounded by, the perceived environment | at the beginning it feels more like it's something in front of me, but somehow with the extending and growing and becoming more three dimensional, having more depth into the distance, I sort of got this feeling that is, it's not something that I'm looking at as much as it's something that is just completely surrounding me. And I'm within it. (P14) |
|  |  | ↖Outside |  |  |  | 13 | A specific value of perceptual position, under which the locus of sense perception is perceived as outside of a perceived environment | it doesn’t feel like we’re in that space, it felt like I was given an insight into that space (P06) |
|  |  | ↖Liminal |  |  |  | 4 | A specific value of perceptual position, under which the locus of sense perception is perceived as transitioning between within and outside a perceived environment | On [the rating of] four I'm physically either moving back or it's receding away from me and there's almost a boulder or door closing and a very visual sense that I'm leaving somewhere and the door is closing. (P07) |
|  | ↳Internalisation |  |  |  |  | 21 | The perceived locus of the emergent DMT experience, or parts of it, in relation to the self-other boundary | (See internal, external, entering) |
|  |  | ↖Internal |  |  |  | 11 | A specific value of internalisation, under which the landscape or parts of it are perceived as within the self | There are always colours around me, and in me. There were things going through me in that moment. (P01) |
|  |  | ↖External |  |  |  | 20 | A specific value of internalisation, under which the landscape or parts of it are perceived as outside the self | at the beginning it feels more like it's something in front of me, but somehow with the extending and growing and becoming more three dimensional, having more depth into the distance, I sort of got this feeling that is, it's not something that I'm looking at as much as it's something that is just completely surrounding me. And I'm within it. (P14) |
|  |  | ↖Entering |  |  |  | 1 | A specific value of internalisation, under which the landscape or parts of it are perceived as extending into the self from outside | It was large and there were these tentacle-like things coming out of it … they were strips like rolling and they were entering me. (P01) |
|  | ↳Self/world distinction |  |  |  |  | 21 | The degree to which an experience featured a clearly distinguished self and environment | (See clearly distinct, indistinct or fused) |
|  |  | ↖Clearly distinct |  |  |  | 17 | A specific value of self/world distinction, under which self and environment are clearly distinguished | They were mostly right in front of my face, and they were like, quite far away, but I had really powerful vision, so I could see them in great detail (P08) |
|  |  | ↖Indistinct or fused |  |  |  | 8 | A specific value of self/world distinction, under which self and environment are fused or otherwise indistinct | [The feeling] started off in the face but it became so overwhelming that I guess I forgot to localise it, or it no longer made sense to localise it to the body. It was very closely related to the visual experience... I actually merged into the scene (P07) |
|  | ↳Identification |  |  |  |  | 21 | The experience of phenomena that are not embodied but nonetheless identified as the self or a representation of the self | (See identified, unidentified) |
|  |  | ↖Identified |  |  |  | 3 | A specific value of identification under which there is a perception of a certain phenomenon as representative of the self, but may not be experienced as part of the self | I broke into tears and I saw these sounds and the meaning of these sounds expressed visually as a character face of myself, layering into this landscape (P01) |
|  |  | ↖Unidentified |  |  |  | 21 | A specific value of identification under which a certain phenomenon is not experienced as representative of the self at all | at the beginning it feels more like it's something in front of me, but somehow with the extending and growing and becoming more three dimensional, having more depth into the distance, I sort of got this feeling that is, it's not something that I'm looking at as much as it's something that is just completely surrounding me. And I'm within it. (P14) |
|  | ↳Interactivity |  |  |  |  | 3 | The perception of a causal relationship between changes in the state of what is perceived to be the self, and the state of what is perceived to be the other | (See indirect, direct) |
|  |  | ↖Indirect |  |  |  | 1 | A specific value of interactivity, under which perceived acts on the part of the self lead to unintentional changes in the structure and / or content of the landscape | I broke into tears and I saw these sounds, and the meaning of these sounds, expressed visually as a character face of myself, layering into this landscape. … [I saw] exactly the same content and material as my expressions. I was certainly shaping this world. (P01) |
|  |  | ↖Direct |  |  |  | 2 | A specific value of interactivity, under which perceived acts on the part of the self lead to intentional changes in the structure and / or content of the landscape | I see this grid of hexagons in the blue and light blue red yellow and teal those hexagons again they are like giant height I can zoom in and out of it and look at this like you look at 3D models (P11) |
|  | ↳Experiential context |  |  |  |  | 11 | The perceived context of experience (distinct from perceptual position as it *contains* the perceptual position) through which the subject apprehends a | (See DMT context only, multiple contexts) |
|  |  | ↖DMT context only |  |  |  | 6 | The experience of an emergent context of experience alone, which has entirely occluded the everyday experiential context (i.e. the experience of the dosing environment is replaced by a DMT environment) | It’s just that everything was starting to be forgotten, and the more I starting to forget things, the more I would have this feeling that I belong to that moment. (P05) |
|  |  | ↖Multiple contexts |  |  |  | 8 | The experience of an emergent experiential context *and* the persisting, everyday experiential context (i.e. simultaneous experience of the DMT environment and the dosing environment) | In my mind I felt things are happening, but I am fully aware I am taking part in a research and that you are there, and probably right next to me... Half of me is up in deep space with the machines, and other half is fully aware, on this planet. (P22) |
|  | ↳Receptive mode |  |  |  |  | 14 | The receptivity of the subject to certain phenomena or the experience as a whole | (See accepted or allowed, resisted) |
|  |  | ↖Accepted or allowed |  |  |  | 11 | A specific value of resistance, under which the subject is accepting (not resisting) certain phenomena, or the experience as a whole | Re-entering it was beautiful. ... there is a conscious willingness to allow the force that is there to do what it does. (P01) |
|  |  | ↖Resisted |  |  |  | 8 | A specific value of resistance, under which the subject is resisting certain phenomena or the experience as a whole | my conscious thought came back into play that it was unpleasant. At those moments when I remembered that I was me and what was going on, I was trying to fight it. (P24) |
|  | ↳Attention |  |  |  |  | 13 | The experience of 'attention' dynamics through which certain phenomena are brought in and out of awareness and/or prominence in awareness (the self being the one who is aware), which may or may not be accompanied by a sense of agency over changes in the scope and target of attention | (See subject to attention, not subject to attention) |
|  |  | ↖Subject to attention |  |  |  | 10 | A specific attentional disposition, under which certain phenomena are centred in awareness | Even when my attention is going off mind-wandering, the stuff is still there in front of my visual field ... It's going on whether I'm looking at it or not. I can chose to think about something else and it will still go on (P07) |
|  |  | ↖Not subject to attention |  |  |  | 8 | A specific attentional disposition, under which certain phenomena are not centred in awareness but may be present in the periphery | Even when my attention is going off mind-wandering, the stuff is still there in front of my visual field ... It's going on whether I'm looking at it or not. I can chose to think about something else and it will still go on (P07) |
|  | ↳Social mode |  |  |  |  | 16 | The interpersonal configuration of an encounter with a perceived presence | (See not engaged, engaged, etc.) |
|  |  | ↖Not engaged |  |  |  | 5 | A specific value of social mode under which a presence is perceived but without any interpersonal engagement | the chanting dudes were in my peripheral vision, they were far away, not really interacting with me, just pulling my attention a little bit (P02) |
|  |  | ↖Engaged |  |  |  | 13 | A specific value of social mode under which a presence is perceived with an interpersonal engagement | PP2: Yellow balls with an orange tinge to the outside of it … they were dancing around and they seemed very mischievous... they definitely had agency |
|  |  |  | ↳Direction of engagement |  |  | 10 | The perceived contribution of the subject in initiating and sustaining an interpersonal engagement with a presence | (See passive, active) |
|  |  |  |  | ↖Passive |  | 13 | A specific value of direction of engagement, under which the the perceived presence is experienced as initiating and sustaining an interpersonal engagement | These entities are there and they’re scanning me (P03) |
|  |  |  |  | ↖Active |  | 2 | A specific value of direction of engagement, under which the the subject is experienced as initiating and/or actively sustaining an interpersonal engagement | I would look more to the monkey for reassurance than the snake (P23) |
|  |  | ↖Communicating |  |  |  | 3 | A specific value of engaged social mode, under which a perceived presence is experienced as communicating | I've got a distinct sense of like, "Okay, you're doing this again." You know, "We were very concerned for you last time. We're not going to be so concerned for you this time." (P17) |
|  |  | ↖Affecting |  |  |  | 2 | A specific value of engaged social mode, under which a perceived presence is experienced as exhibiting behaviours intended to cause an affective response in the subject (e.g.: taunting, reassuring) | The faces are …fucking with me in a very playful way .. like pulling their tongues out (P03) |
|  |  | ↖Guiding |  |  |  | 3 | A specific value of engaged social mode, under which a perceived presence is experienced as moving the self and perceptual position from one spatial location to another | I know I have to go with it… Because its with me, its in charge of where I’m going (P23) |
|  |  | ↖Manipulating |  |  |  | 2 | A specific value of engaged social mode, under which a perceived presence is experienced as physically manipulating the subject's body | these entities just spreading my body, smearing me with the environment (P03) |
|  |  | ↖Observing |  |  |  | 3 | A specific value of engaged social mode, under which a perceived presence is experienced as actively observing the subject | These entities are there and they’re scanning me (P03) |
|  |  | ↖Nurturing / healing |  |  |  | 3 | A specific value of engaged social mode, under which a perceived presence is experienced as directing a healing / nurturing act or intention toward the subject | They were entering me. Then my whole being felt filled with completeness and then I realized that 'this is it.' 'I am here.' 'This is it.' I felt so welcomed and so embraced and so, so nurtured by this. I knew that’s it, I am here. Thank you for doing this and showing this. (P01) |
|  |  | ↖Presenting / showing |  |  |  | 4 | A specific value of engaged social mode, under which a perceived presence is revealing or otherwise directing the subject's attention towards a certain phenomenon | I felt like they were showing something to me or guiding me. So, they were with me, trying to show me something, all these feelings that can ever be. (P24) |
